# Crystal structure of bis­{μ-4-methyl-*N*′-[3-(oxido­imino)­butan-2-yl­idene]benzene­sulfono­hydrazidato}bis­[(dimethyl sulfoxide-κ*O*)copper(II)]

**DOI:** 10.1107/S1600536814016651

**Published:** 2014-08-01

**Authors:** Diego Pereira Siqueira, Maria Carolina Bulhosa Siqueira, Vanessa Carratu Gervini, Leandro Bresolin, Adriano Bof de Oliveira

**Affiliations:** a Escola de Química e Alimentos, Universidade Federal do Rio Grande, Av. Itália km 08, Campus Carreiros, 96203-900, Rio Grande, RS, Brazil; b Departamento de Química, Universidade Federal de Sergipe, Av. Marechal Rondon s/n, Campus, 49100-000, São Cristóvão, SE, Brazil

**Keywords:** crystal structure, hy­droxy­imino-tosyl­hydrazone derivative, Cu^II^ dimer, π–π stacking

## Abstract

In the title compound, [Cu_2_(C_11_H_13_N_3_O_3_S)_2_(C_2_H_6_OS)_2_], the Cu^II^ cation is *N*,*N*′,*O*-chelated by a deprotonated hy­droxy­imino-tosyl­hydrazone ligand and coordinated by a dimethyl sulfoxide mol­ecule. One O atom from the adjacent hy­droxy­imino-tosyl­hydrazone ligand bridges the Cu^II^ cation, forming the centrosymmetric dimeric complex. The cation is in an overall distorted N_2_O_3_ square-pyramidal coordination environment. The methyl­benzene ring is twisted with respect to the hydrazine fragment, with a dihedral angle of 89.54 (9)° between the planes. An intra­molecular C—H⋯O hydrogen bond occurs. In the crystal, mol­ecules are linked by weak C—H⋯O and C—H⋯S inter­actions. Weak π–π stacking is also observed between parallel benzene rings of adjacent mol­ecules, the centroid–centroid distance being 3.9592 (17) Å.

## Related literature   

For the synthesis and applications of hy­droxy­imino-tosyl­hydrazones as complexing agents, see: Beger *et al.* (1991 ▶). For the crystal structure of the 4-methyl-*N*′-[3-(hy­droxy­imino)­butan-2-yl­idene]benzene­sulfono­hydrazide ligand, see: Bulhosa *et al.* (2012 ▶).
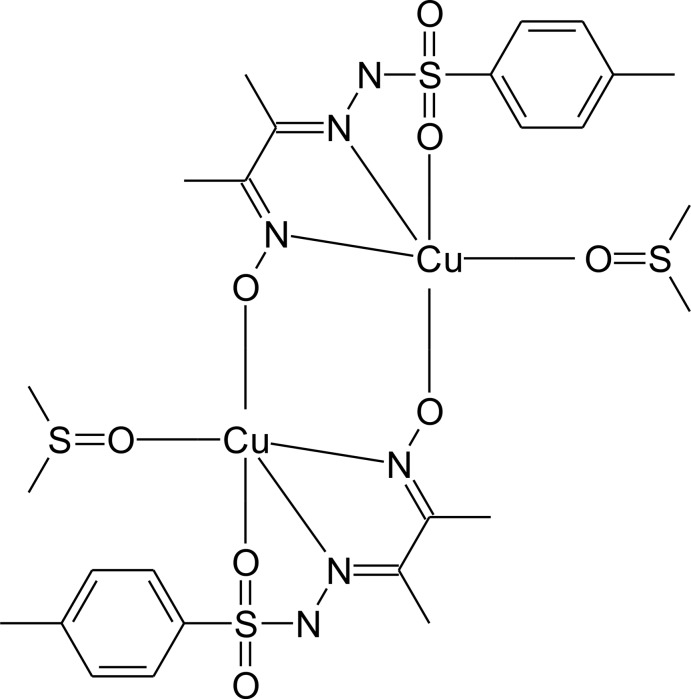


## Experimental   

### Crystal data  

[Cu_2_(C_11_H_13_N_3_O_3_S)_2_(C_2_H_6_OS)_2_]
*M* *_r_* = 817.95
Triclinic, 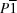

*a* = 7.8097 (3) Å
*b* = 8.4670 (3) Å
*c* = 15.1586 (6) Å
α = 74.656 (2)°
β = 75.955 (2)°
γ = 65.042 (2)°
*V* = 866.47 (6) Å^3^
*Z* = 1
Mo *K*α radiation
μ = 1.52 mm^−1^
*T* = 293 K
0.61 × 0.28 × 0.07 mm

### Data collection  

Bruker APEXII CCD diffractometer
Absorption correction: multi-scan (*SADABS*; Bruker, 2005 ▶) *T*_min_ = 0.457, *T*_max_ = 0.901
5765 measured reflections
4054 independent reflections
3366 reflections with *I* > 2σ(*I*)
*R*_int_ = 0.015

### Refinement  

*R*[*F* ^2^ > 2σ(*F* ^2^)] = 0.032
*wR*(*F* ^2^) = 0.091
*S* = 1.04
4054 reflections
208 parameters
H-atom parameters constrained
Δρ_max_ = 0.38 e Å^−3^
Δρ_min_ = −0.42 e Å^−3^

### 

Data collection: *APEX2* (Bruker, 2005 ▶); cell refinement: *SAINT* (Bruker, 2005 ▶); data reduction: *SAINT*; program(s) used to solve structure: *SHELXTL* (Sheldrick, 2008 ▶); program(s) used to refine structure: *SHELXTL*; molecular graphics: *SHELXTL*; software used to prepare material for publication: *SHELXTL*.

## Supplementary Material

Crystal structure: contains datablock(s) I, global. DOI: 10.1107/S1600536814016651/xu5805sup1.cif

**Fig. 1.**
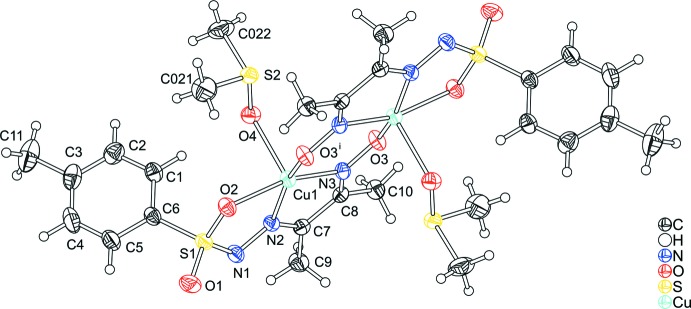
: The molecular structure of the title compound with labeling and displacement ellipsoids drawn at the 40% probability level showing the dimeric structure. Symmetry code: (i)-x + 1,-y + 1,-z + 1

**Fig. 2.**
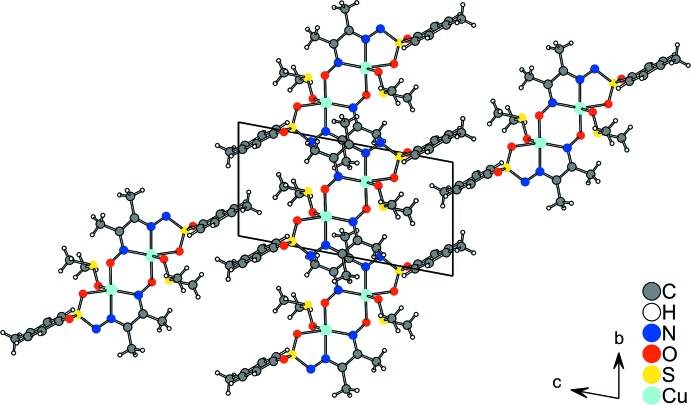
: Molecules of the title compound arranged along b-axis showing the column of the aromatic rings with very weak π–π interactions.

Structure factors: contains datablock(s) I. DOI: 10.1107/S1600536814016651/xu5805Isup2.hkl

x y z . DOI: 10.1107/S1600536814016651/xu5805fig1.tif
The mol­ecular structure of the title compound with labeling and displacement ellipsoids drawn at the 40% probability level showing the dimeric structure. Symmetry code: (i)-*x* + 1,-*y* + 1,-*z* + 1

b . DOI: 10.1107/S1600536814016651/xu5805fig2.tif
Mol­ecules of the title compound arranged along *b*-axis showing the column of the aromatic rings with very weak π–π inter­actions.

CCDC reference: 1014769

Additional supporting information:  crystallographic information; 3D view; checkCIF report

## Figures and Tables

**Table 1:** Selected bond lengths (Å)

Cu1—N2	1.9580 (19)
Cu1—N3	1.9728 (18)
Cu1—O2	2.0970 (16)
Cu1—O3^i^	1.8798 (16)
Cu1—O4	2.2517 (17)

Symmetry code: (i) 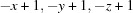.

**Table 2:** Hydrogen-bond geometry (Å, °)

*D*—H⋯*A*	*D*—H	H⋯*A*	*D*⋯*A*	*D*—H⋯*A*
C1—H1⋯O4	0.93	2.39	3.299 (3)	166
C2—H2⋯O1^ii^	0.93	2.57	3.430 (4)	154
C9—H9*A*⋯S2^iii^	0.96	2.75	3.693 (3)	166
C10—H10*C*⋯O1^iv^	0.96	2.47	3.415 (4)	166

Symmetry codes: (ii) 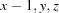; (iii) 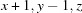; (iv) 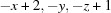.

## supplementary crystallographic information

### S2. Structural commentary

Hy­droxy­imino-tosyl­hydrazone derivatives are N,O-donors that show an application as complexing agents (Beger *et al.*, 1991). In the crystal structure of the title compound the Cu^II^ cations are five-coordinated by one crystallographically independent deprotonated hy­droxy­imino-tosyl­hydrazone derivative, one DMSO molecule and one O-atom from a second, symmetry generated, hy­droxy­imino-tosyl­hydrazone derivative into dimers (Fig. 1). The metal centres are in a slightly distorted pyramidal environment. The aromatic ring and the N1/N2/C7/C8/N3/O3-fragment angle amount to 89,54 (09)°. In this complex molecule significant structural changes of the N–O and N–N bonds. For the uncoordinated ligand the N–O and N–N bonds distances amount to 1.4084 (16) Å and 1.3807 (16) Å. These distances indicate the double bond character for the N–N and the single bond character for the N–O bond (Bulhosa *et al.*, 2012). In contrast, in the title compound, the acidic hydrogen of the hydrazine fragment is removed and the negative charge is delocalized over the N–N–C–C–N–O fragment. Therefore, N–N and N–O distances amount to 1.367 (3) Å and 1.343 (2) Å. Additionally, the complexes are linked by N–O bridges into dimers (Fig. 2). Finally, the dimers are arranged along the *b*-axis with very weak π–π inter­actions.

### S5. Synthesis and crystallization

Starting materials were commercially available and were used without further purification. The ligand synthesis was adapted from a procedure reported previously and its structure is already published (Bulhosa *et al.*, 2012). *N*'-[3-(Hy­droxy­imino)­butan- 2-yl­idene]-4-methyl­benzene-1-sulfono­hydrazide was dissolved in methanol (2 mmol/40 mL) with stirring maintained for 30 min and deprotonated with sodium, while the solution turns yellow. At the same time, a solution of copper(II) acetate monohydrate (1 mmol/40 mL) in methanol was prepared under continuous stirring. A mixture of both solutions was maintained with stirring at room temperature for 6 h. The methanol was removed by evaporation and crystals suitable for X-ray diffraction were obtained in DMSO by the slow evaporation of the solvent.

### S6. Refinement

H atoms attached to C atoms were positioned with idealized geometry and were refined isotropically with *U*_iso_(H) set to 1.2 times *U*_eq_(C) for the aromatic and 1.5 times *U*_eq_(C) for methyl H atoms using a riding model with C—H = 0.93 Å and C—H = 0.96 Å, respectively.

### Crystal data

**Table:**

[Cu_2_(C_11_H_13_N_3_O_3_S)_2_(C_2_H_6_OS)_2_]	*Z* = 1
*M**_r_* = 817.95	*F*(000) = 422
Triclinic, *P*1	*D*_x_ = 1.568 Mg m^−^^3^
Hall symbol: -P 1	Mo *K*α radiation, λ = 0.71073 Å
*a* = 7.8097 (3) Å	Cell parameters from 7693 reflections
*b* = 8.4670 (3) Å	θ = 2.8–28.1°
*c* = 15.1586 (6) Å	µ = 1.52 mm^−^^1^
α = 74.656 (2)°	*T* = 293 K
β = 75.955 (2)°	Block, black
γ = 65.042 (2)°	0.61 × 0.28 × 0.07 mm
*V* = 866.47 (6) Å^3^	

### Data collection

**Table:**

Bruker APEXII CCD diffractometer	4054 independent reflections
Radiation source: fine-focus sealed tube, Bruker Kappa CCD	3366 reflections with *I* > 2σ(*I*)
Graphite monochromator	*R*_int_ = 0.015
φ and ω scans	θ_max_ = 28.4°, θ_min_ = 2.7°
Absorption correction: multi-scan (*SADABS*; Bruker, 2005)	*h* = −10→10
*T*_min_ = 0.457, *T*_max_ = 0.901	*k* = −11→7
5765 measured reflections	*l* = −20→18

### Refinement

**Table:**

Refinement on *F*^2^	Primary atom site location: structure-invariant direct methods
Least-squares matrix: full	Secondary atom site location: difference Fourier map
*R*[*F*^2^ > 2σ(*F*^2^)] = 0.032	Hydrogen site location: inferred from neighbouring sites
*wR*(*F*^2^) = 0.091	H-atom parameters constrained
*S* = 1.04	*w* = 1/[σ^2^(*F*_o_^2^) + (0.0467*P*)^2^ + 0.3887*P*] where *P* = (*F*_o_^2^ + 2*F*_c_^2^)/3
4054 reflections	(Δ/σ)_max_ < 0.001
208 parameters	Δρ_max_ = 0.38 e Å^−^^3^
0 restraints	Δρ_min_ = −0.42 e Å^−^^3^

### Special details

**Table:**

Geometry. All e.s.d.'s (except the e.s.d. in the dihedral angle between two l.s. planes) are estimated using the full covariance matrix. The cell e.s.d.'s are taken into account individually in the estimation of e.s.d.'s in distances, angles and torsion angles; correlations between e.s.d.'s in cell parameters are only used when they are defined by crystal symmetry. An approximate (isotropic) treatment of cell e.s.d.'s is used for estimating e.s.d.'s involving l.s. planes.
Refinement. Refinement of *F*^2^ against ALL reflections. The weighted *R*-factor *wR* and goodness of fit *S* are based on *F*^2^, conventional *R*-factors *R* are based on *F*, with *F* set to zero for negative *F*^2^. The threshold expression of *F*^2^ > σ(*F*^2^) is used only for calculating *R*-factors(gt) *etc*. and is not relevant to the choice of reflections for refinement. *R*-factors based on *F*^2^ are statistically about twice as large as those based on *F*, and *R*- factors based on ALL data will be even larger.

### Fractional atomic coordinates and isotropic or equivalent isotropic displacement parameters (Å^2^)

**Table:**

	*x*	*y*	*z*	*U*_iso_*/*U*_eq_	
Cu1	0.58200 (4)	0.31552 (3)	0.593265 (18)	0.02826 (9)	
S1	0.77302 (8)	0.04077 (8)	0.74973 (4)	0.03261 (14)	
S2	0.14080 (8)	0.50296 (8)	0.67789 (4)	0.03775 (15)	
O2	0.6646 (2)	0.2330 (2)	0.72499 (11)	0.0355 (4)	
N2	0.7236 (3)	0.0618 (2)	0.59334 (13)	0.0287 (4)	
C10	0.6295 (4)	0.1222 (3)	0.35609 (16)	0.0370 (5)	
H10A	0.5581	0.2316	0.3194	0.056*	
H10B	0.5663	0.0413	0.3685	0.056*	
H10C	0.7556	0.0715	0.3231	0.056*	
C7	0.7440 (3)	0.0142 (3)	0.51572 (15)	0.0293 (4)	
O4	0.2837 (2)	0.3281 (2)	0.65248 (12)	0.0386 (4)	
O1	0.9442 (3)	−0.0107 (3)	0.78807 (13)	0.0480 (5)	
N1	0.8225 (3)	−0.0535 (3)	0.66265 (13)	0.0361 (4)	
O3	0.4716 (2)	0.4478 (2)	0.40297 (11)	0.0346 (4)	
N3	0.5685 (3)	0.3140 (2)	0.46524 (12)	0.0281 (4)	
C8	0.6430 (3)	0.1565 (3)	0.44490 (15)	0.0271 (4)	
C2	0.3225 (4)	−0.0775 (4)	0.88982 (19)	0.0486 (7)	
H2	0.1983	−0.0485	0.8810	0.058*	
C1	0.4370 (4)	−0.0029 (4)	0.82421 (17)	0.0400 (6)	
H1	0.3897	0.0761	0.7719	0.048*	
C6	0.6226 (3)	−0.0460 (3)	0.83647 (15)	0.0332 (5)	
C9	0.8650 (4)	−0.1691 (3)	0.49726 (19)	0.0417 (6)	
H9A	0.9189	−0.2428	0.5517	0.062*	
H9B	0.9659	−0.1646	0.4470	0.062*	
H9C	0.7878	−0.2174	0.4813	0.062*	
C3	0.3885 (5)	−0.1948 (4)	0.96864 (19)	0.0526 (7)	
C5	0.6905 (4)	−0.1601 (4)	0.91562 (18)	0.0471 (6)	
H5	0.8141	−0.1876	0.9251	0.057*	
C4	0.5723 (5)	−0.2326 (4)	0.9805 (2)	0.0583 (8)	
H4	0.6181	−0.3091	1.0337	0.070*	
C021	0.1846 (5)	0.5082 (5)	0.7870 (2)	0.0674 (9)	
H02A	0.3027	0.5237	0.7789	0.101*	
H02B	0.1927	0.3987	0.8286	0.101*	
H02C	0.0821	0.6049	0.8122	0.101*	
C022	−0.0802 (4)	0.4738 (5)	0.7154 (3)	0.0650 (9)	
H02D	−0.1256	0.4685	0.6633	0.097*	
H02E	−0.1723	0.5718	0.7436	0.097*	
H02F	−0.0618	0.3656	0.7597	0.097*	
C11	0.2614 (6)	−0.2742 (5)	1.0403 (3)	0.0812 (12)	
H11A	0.1395	−0.2329	1.0205	0.122*	
H11B	0.2444	−0.2393	1.0982	0.122*	
H11C	0.3194	−0.4011	1.0480	0.122*	

### Atomic displacement parameters (Å^2^)

**Table:**

	*U*^11^	*U*^22^	*U*^33^	*U*^12^	*U*^13^	*U*^23^
Cu1	0.03260 (15)	0.02483 (15)	0.02657 (15)	−0.00907 (11)	−0.00598 (10)	−0.00532 (10)
S1	0.0312 (3)	0.0331 (3)	0.0312 (3)	−0.0074 (2)	−0.0101 (2)	−0.0054 (2)
S2	0.0296 (3)	0.0328 (3)	0.0422 (3)	−0.0086 (2)	−0.0041 (2)	0.0001 (2)
O2	0.0420 (9)	0.0315 (9)	0.0331 (8)	−0.0111 (7)	−0.0110 (7)	−0.0062 (7)
N2	0.0290 (9)	0.0258 (9)	0.0288 (9)	−0.0084 (7)	−0.0051 (7)	−0.0038 (7)
C10	0.0443 (13)	0.0342 (13)	0.0342 (12)	−0.0130 (10)	−0.0068 (10)	−0.0115 (10)
C7	0.0272 (10)	0.0285 (11)	0.0315 (11)	−0.0107 (9)	−0.0015 (8)	−0.0071 (9)
O4	0.0341 (9)	0.0360 (9)	0.0434 (10)	−0.0108 (7)	−0.0056 (7)	−0.0080 (7)
O1	0.0360 (9)	0.0588 (12)	0.0493 (11)	−0.0131 (9)	−0.0180 (8)	−0.0081 (9)
N1	0.0388 (11)	0.0311 (10)	0.0289 (9)	−0.0027 (8)	−0.0078 (8)	−0.0057 (8)
O3	0.0486 (10)	0.0259 (8)	0.0310 (8)	−0.0119 (7)	−0.0166 (7)	−0.0023 (6)
N3	0.0315 (9)	0.0268 (9)	0.0270 (9)	−0.0129 (7)	−0.0048 (7)	−0.0034 (7)
C8	0.0265 (10)	0.0282 (11)	0.0277 (10)	−0.0122 (8)	−0.0009 (8)	−0.0071 (8)
C2	0.0486 (15)	0.0597 (18)	0.0432 (15)	−0.0256 (14)	−0.0016 (12)	−0.0154 (13)
C1	0.0441 (14)	0.0450 (14)	0.0322 (12)	−0.0163 (11)	−0.0121 (10)	−0.0049 (11)
C6	0.0394 (12)	0.0306 (12)	0.0276 (11)	−0.0080 (9)	−0.0097 (9)	−0.0069 (9)
C9	0.0451 (14)	0.0316 (12)	0.0417 (14)	−0.0051 (10)	−0.0072 (11)	−0.0117 (11)
C3	0.073 (2)	0.0493 (17)	0.0369 (14)	−0.0293 (15)	0.0038 (13)	−0.0115 (12)
C5	0.0512 (16)	0.0452 (15)	0.0355 (13)	−0.0083 (12)	−0.0157 (11)	−0.0011 (11)
C4	0.081 (2)	0.0475 (17)	0.0336 (14)	−0.0170 (16)	−0.0146 (14)	0.0048 (12)
C021	0.071 (2)	0.070 (2)	0.057 (2)	−0.0141 (18)	−0.0073 (16)	−0.0295 (17)
C022	0.0335 (14)	0.060 (2)	0.091 (3)	−0.0199 (14)	0.0026 (15)	−0.0057 (18)
C11	0.111 (3)	0.077 (3)	0.057 (2)	−0.055 (2)	0.020 (2)	−0.0123 (19)

### Geometric parameters (Å, º)

**Table:**

Cu1—N2	1.9580 (19)	C2—C1	1.378 (4)
Cu1—N3	1.9728 (18)	C2—C3	1.385 (4)
Cu1—O2	2.0970 (16)	C2—H2	0.9300
Cu1—O3^i^	1.8798 (16)	C1—C6	1.385 (3)
Cu1—O4	2.2517 (17)	C1—H1	0.9300
S1—O1	1.4376 (18)	C6—C5	1.385 (3)
S1—O2	1.4745 (17)	C9—H9A	0.9600
S1—N1	1.606 (2)	C9—H9B	0.9600
S1—C6	1.765 (2)	C9—H9C	0.9600
S2—O4	1.5114 (18)	C3—C4	1.379 (5)
S2—C022	1.781 (3)	C3—C11	1.507 (4)
S2—C021	1.783 (3)	C5—C4	1.384 (4)
N2—C7	1.295 (3)	C5—H5	0.9300
N2—N1	1.367 (3)	C4—H4	0.9300
C10—C8	1.486 (3)	C021—H02A	0.9600
C10—H10A	0.9600	C021—H02B	0.9600
C10—H10B	0.9600	C021—H02C	0.9600
C10—H10C	0.9600	C022—H02D	0.9600
C7—C8	1.467 (3)	C022—H02E	0.9600
C7—C9	1.498 (3)	C022—H02F	0.9600
O3—N3	1.343 (2)	C11—H11A	0.9600
O3—Cu1^i^	1.8798 (16)	C11—H11B	0.9600
N3—C8	1.299 (3)	C11—H11C	0.9600
			
O3^i^—Cu1—N2	160.52 (8)	C1—C2—H2	119.3
O3^i^—Cu1—N3	105.85 (7)	C3—C2—H2	119.3
N2—Cu1—N3	81.34 (8)	C2—C1—C6	119.7 (2)
O3^i^—Cu1—O2	90.50 (6)	C2—C1—H1	120.1
N2—Cu1—O2	80.08 (7)	C6—C1—H1	120.1
N3—Cu1—O2	160.90 (7)	C1—C6—C5	119.9 (2)
O3^i^—Cu1—O4	95.33 (7)	C1—C6—S1	119.73 (18)
N2—Cu1—O4	101.95 (7)	C5—C6—S1	120.4 (2)
N3—Cu1—O4	96.11 (7)	C7—C9—H9A	109.5
O2—Cu1—O4	92.01 (7)	C7—C9—H9B	109.5
O1—S1—O2	116.12 (11)	H9A—C9—H9B	109.5
O1—S1—N1	109.45 (11)	C7—C9—H9C	109.5
O2—S1—N1	110.60 (10)	H9A—C9—H9C	109.5
O1—S1—C6	105.75 (11)	H9B—C9—H9C	109.5
O2—S1—C6	107.02 (11)	C4—C3—C2	117.8 (3)
N1—S1—C6	107.42 (11)	C4—C3—C11	121.2 (3)
O4—S2—C022	105.09 (14)	C2—C3—C11	121.0 (3)
O4—S2—C021	106.15 (14)	C4—C5—C6	119.1 (3)
C022—S2—C021	98.59 (18)	C4—C5—H5	120.4
S1—O2—Cu1	114.45 (9)	C6—C5—H5	120.4
C7—N2—N1	120.75 (19)	C3—C4—C5	121.9 (3)
C7—N2—Cu1	114.72 (15)	C3—C4—H4	119.0
N1—N2—Cu1	123.60 (15)	C5—C4—H4	119.0
C8—C10—H10A	109.5	S2—C021—H02A	109.5
C8—C10—H10B	109.5	S2—C021—H02B	109.5
H10A—C10—H10B	109.5	H02A—C021—H02B	109.5
C8—C10—H10C	109.5	S2—C021—H02C	109.5
H10A—C10—H10C	109.5	H02A—C021—H02C	109.5
H10B—C10—H10C	109.5	H02B—C021—H02C	109.5
N2—C7—C8	114.58 (19)	S2—C022—H02D	109.5
N2—C7—C9	123.5 (2)	S2—C022—H02E	109.5
C8—C7—C9	121.8 (2)	H02D—C022—H02E	109.5
S2—O4—Cu1	116.15 (10)	S2—C022—H02F	109.5
N2—N1—S1	110.24 (15)	H02D—C022—H02F	109.5
N3—O3—Cu1^i^	120.90 (13)	H02E—C022—H02F	109.5
C8—N3—O3	117.04 (18)	C3—C11—H11A	109.5
C8—N3—Cu1	113.71 (15)	C3—C11—H11B	109.5
O3—N3—Cu1	128.55 (14)	H11A—C11—H11B	109.5
N3—C8—C7	115.07 (19)	C3—C11—H11C	109.5
N3—C8—C10	122.8 (2)	H11A—C11—H11C	109.5
C7—C8—C10	122.1 (2)	H11B—C11—H11C	109.5
C1—C2—C3	121.5 (3)		
			
O1—S1—O2—Cu1	132.54 (11)	O3^i^—Cu1—N3—C8	165.37 (15)
N1—S1—O2—Cu1	7.05 (14)	N2—Cu1—N3—C8	3.91 (15)
C6—S1—O2—Cu1	−109.67 (11)	O2—Cu1—N3—C8	17.4 (3)
O3^i^—Cu1—O2—S1	−164.58 (11)	O4—Cu1—N3—C8	−97.30 (15)
N2—Cu1—O2—S1	−1.72 (10)	O3^i^—Cu1—N3—O3	−24.6 (2)
N3—Cu1—O2—S1	−15.2 (3)	N2—Cu1—N3—O3	173.91 (18)
O4—Cu1—O2—S1	100.06 (11)	O2—Cu1—N3—O3	−172.62 (17)
O3^i^—Cu1—N2—C7	−112.5 (2)	O4—Cu1—N3—O3	72.70 (17)
N3—Cu1—N2—C7	0.93 (15)	O3—N3—C8—C7	−178.83 (17)
O2—Cu1—N2—C7	−174.63 (16)	Cu1—N3—C8—C7	−7.6 (2)
O4—Cu1—N2—C7	95.40 (16)	O3—N3—C8—C10	1.8 (3)
O3^i^—Cu1—N2—N1	56.5 (3)	Cu1—N3—C8—C10	173.05 (16)
N3—Cu1—N2—N1	170.01 (18)	N2—C7—C8—N3	8.5 (3)
O2—Cu1—N2—N1	−5.55 (17)	C9—C7—C8—N3	−169.7 (2)
O4—Cu1—N2—N1	−95.52 (17)	N2—C7—C8—C10	−172.10 (19)
N1—N2—C7—C8	−174.51 (18)	C9—C7—C8—C10	9.6 (3)
Cu1—N2—C7—C8	−5.1 (2)	C3—C2—C1—C6	0.4 (4)
N1—N2—C7—C9	3.7 (3)	C2—C1—C6—C5	−1.7 (4)
Cu1—N2—C7—C9	173.13 (18)	C2—C1—C6—S1	176.8 (2)
C022—S2—O4—Cu1	176.67 (15)	O1—S1—C6—C1	173.2 (2)
C021—S2—O4—Cu1	−79.50 (17)	O2—S1—C6—C1	48.8 (2)
O3^i^—Cu1—O4—S2	−1.22 (11)	N1—S1—C6—C1	−70.0 (2)
N2—Cu1—O4—S2	169.76 (11)	O1—S1—C6—C5	−8.3 (2)
N3—Cu1—O4—S2	−107.83 (11)	O2—S1—C6—C5	−132.7 (2)
O2—Cu1—O4—S2	89.47 (11)	N1—S1—C6—C5	108.5 (2)
C7—N2—N1—S1	179.00 (16)	C1—C2—C3—C4	1.1 (4)
Cu1—N2—N1—S1	10.5 (2)	C1—C2—C3—C11	179.4 (3)
O1—S1—N1—N2	−139.82 (16)	C1—C6—C5—C4	1.5 (4)
O2—S1—N1—N2	−10.66 (19)	S1—C6—C5—C4	−177.0 (2)
C6—S1—N1—N2	105.82 (17)	C2—C3—C4—C5	−1.4 (5)
Cu1^i^—O3—N3—C8	−162.43 (15)	C11—C3—C4—C5	−179.6 (3)
Cu1^i^—O3—N3—Cu1	27.9 (2)	C6—C5—C4—C3	0.1 (5)

Symmetry code: (i) −*x*+1, −*y*+1, −*z*+1.

### Hydrogen-bond geometry (Å, º)

**Table:**

*D*—H···*A*	*D*—H	H···*A*	*D*···*A*	*D*—H···*A*
C1—H1···O4	0.93	2.39	3.299 (3)	166
C2—H2···O1^ii^	0.93	2.57	3.430 (4)	154
C9—H9*A*···S2^iii^	0.96	2.75	3.693 (3)	166
C10—H10*C*···O1^iv^	0.96	2.47	3.415 (4)	166

Symmetry codes: (ii) *x*−1, *y*, *z*; (iii) *x*+1, *y*−1, *z*; (iv) −*x*+2, −*y*, −*z*+1.
