# Supplementary figures and images for: Crystal structure of bis­{μ-4-methyl-N′-[3-(oxido­imino)­butan-2-yl­idene]benzene­sulfono­hydrazidato}bis­[(dimethyl sulfoxide-κO)copper(II)]
Source: Acta Crystallogr Sect E Struct Rep Online. 2014 Aug 1;70(Pt 9):m316–7. doi: 10.1107/S1600536814016651 (PMC4186093; doi:10.1107/S1600536814016651)

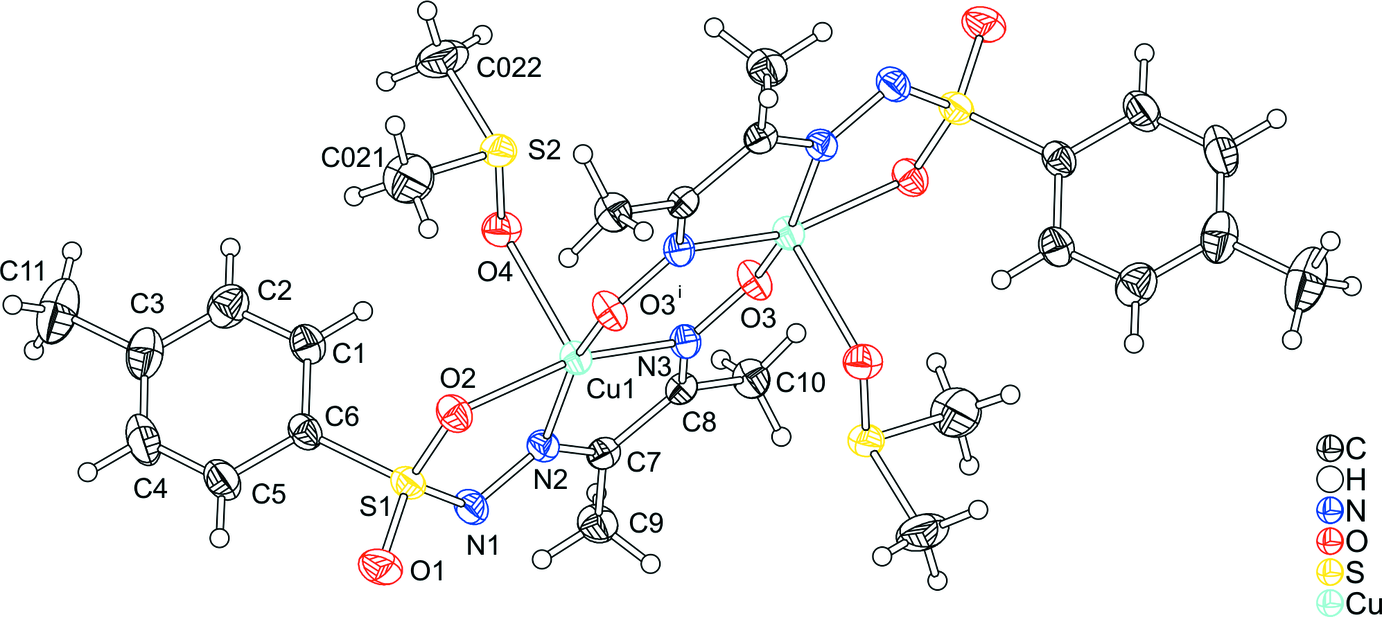

Supplement: Supplementary file 3 [file e-70-0m316-fig1.tif]

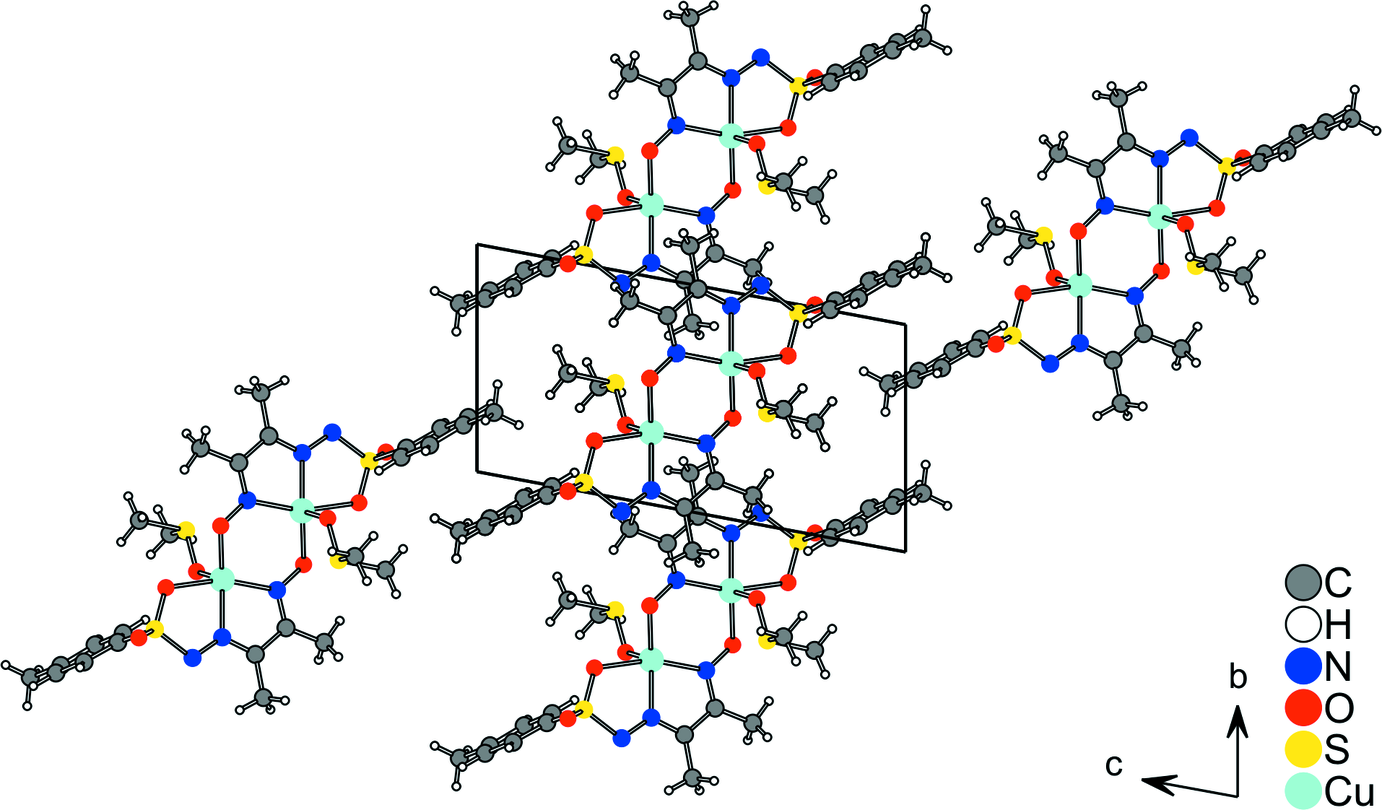

Supplement: Supplementary file 4 [file e-70-0m316-fig2.tif]
